# Identifying Adverse Drug Events in Clinical Text Using Fine-Tuned Clinical Language Models: Machine Learning Study

**DOI:** 10.2196/71949

**Published:** 2025-09-11

**Authors:** Elizaveta Kopacheva, Aron Henriksson, Hercules Dalianis, Tora Hammar, Alisa Lincke

**Affiliations:** 1 Department of Computer Science and Media Technology, Faculty of Technology, Linnaeus University, Universitetsplatsen 1, Växjö, 352 52, Sweden, 46 737033730; 2 Department of Computer and Systems Sceince, Stockholm University, Kista, Sweden; 3 eHealth Institute, Department of Medicine and Optometry, Faculty of Health and Life Sciences, Linnaeus University, Växjö, Sweden

**Keywords:** electronical health records, adverse drug events, domain-specific language models, BERT, SweDeClin-BERT

## Abstract

**Background:**

Medications are essential for health care but can cause adverse drug events (ADEs), which are harmful and sometimes fatal. Detecting ADEs is a challenging task because they are often not documented in the structured data of electronic health records (EHRs). There is a need for automatically extracting ADE-related information from clinical notes, as manual review is labor-intensive and time-consuming.

**Objective:**

This study aims to fine-tune the pretrained clinical language model, Swedish Deidentified Clinical Bidirectional Encoder Representations from Transformers (SweDeClin-BERT), for medical named entity recognition (NER) and relation extraction (RE) tasks, and to implement an integrated NER-RE approach to more effectively identify ADEs in clinical notes from clinical units in Sweden. The performance of this approach is compared with our previous machine learning method, which used conditional random fields (CRFs) and random forest (RF).

**Methods:**

A subset of clinical notes from the Stockholm EPR (Electronic Patient Record) Corpus, dated 2009‐2010, containing suspected ADEs based on *International Classification of Diseases, 10th Revision* (*ICD-10*) codes in the A.1 and A.2 categories was randomly sampled. These notes were annotated by a physician with ADE-related entities and relations following the ADE annotation guidelines. We fine-tuned the SweDeClin-BERT model for the NER and RE tasks and implemented an integrated NER-RE pipeline to extract entities and relationships from clinical notes. The models were evaluated using 395 clinical notes from clinical units in Sweden. The NER-RE pipeline was then applied to classify the clinical notes as containing or not containing ADEs. In addition, we conducted an error analysis to better understand the model’s behavior and to identify potential areas for improvement.

**Results:**

In total, 62% of notes contained an explicit description of an ADE, indicating that an ADE-related *ICD-10* code alone does not ensure detailed event documentation. The fine-tuned SweDeClin-BERT model achieved an *F*_1_-score of 0.845 for NER and 0.81 for RE task, outperforming the baseline models (CRFs for NER and random forests for RE). In particular, the RE task showed a 53% improvement in macro-average *F*_1_-score compared to the baseline. The integrated NER-RE pipeline achieved an overall *F*_1_-score of 0.81.

**Conclusions:**

Using a domain-specific language model like SweDeClin-BERT for detecting ADEs in clinical notes demonstrates improved classification performance (0.77 in strict and 0.81 in relaxed mode) compared to conventional machine learning models like CRFs and RF. The proposed fine-tuned ADE model requires further refinement and evaluation on annotated clinical notes from another hospital to evaluate the model’s generalizability. In addition, the annotation guidelines should be revised, as there is an overlap of words between the Finding and Disorder entity categories, which were not consistently distinguished by the annotators. Furthermore, future work should address the handling of compound words and split entities to better capture context in the Swedish language.

## Introduction

Medications are an essential part of health care; they improve the lives of many but can also cause unwanted effects. Adverse drug events (ADEs) are harmful events in patients that are related to the use of a medication, including adverse drug reactions (ADRs, also called “side effects”). ADEs are common and cause hardship or even death to patients and a significant cost to society [1]. However, numbers on the prevalence and cost differ extensively between studies in different settings and depending on how they are measured [2,3]. One systematic review estimated that 19% of patients are affected by ADEs during hospitalization [2]. Another systematic review estimated that rates of readmission to hospitals due to medications varied from 3% to 64%, with the majority of them being preventable [4]. Among outpatients, it has been estimated that 2% have preventable ADRs [5]. The widespread use of electronic health records (EHRs) provides large amounts of data that hold great potential for pharmacovigilance and monitoring of ADEs in the population [6]. Information about actual or suspected ADEs can be documented in the EHR in different ways, either as diagnosis codes (structured data) or written in free text (unstructured data). In Sweden, ADEs can be detected using clinical decision support systems (CDSS) such as the Janusmed knowledge databases [7] and Bikt (part of the Swedish Information Services [SIL]) [8], which operate on structured data like medication lists. However, these systems rely on rule-based algorithms and substance-level drug knowledge, often generating an excessive number of alerts—leading to alert fatigue—and are currently integrated into only a limited number of EHR systems. All suspected ADEs should be reported to national authorities, such as the Swedish Medical Products Agency, or by conducting occasional manual chart reviews. There is currently no routine or automated method for monitoring and extracting suspected ADEs directly from unstructured clinical notes in EHRs in Sweden.

Recent advances in technological development and artificial intelligence (AI) offer improved possibilities for analyzing large amounts of data, which is useful in pharmacovigilance, among other [9,10]. Over the years, researchers have dedicated extensive efforts to develop effective methods and algorithms to detect and recognize ADEs within the information stored in both structured data (like diagnosis codes in EHRs) and unstructured data, such as clinical notes, which is more challenging. Research in the field has evolved alongside advancements in natural language processing (NLP) and machine learning techniques. Specifically, earlier approaches [11,12] are knowledge-based approaches that rely on predefined rules and medical dictionaries to extract ADEs from clinical notes. Later on, improvements in NLP leveraged linguistic text processing, and medical ontologies began to be widely used. More recent studies [13-16] are data-driven approaches that have relied on supervised machine learning and, more often, deep learning techniques for better classification of ADEs using annotated clinical notes. Recent advancements in AI, such as large language models (LLMs), transformer-based models, knowledge graphs, and transfer learning, have demonstrated state-of-the-art performance in identifying ADEs in clinical notes [17-21]. LLM is a sub-branch of the NLP branch of AI designed to understand, generate, and manipulate text for a variety of language-related tasks [22].

Previous research on detecting ADEs from clinical texts shows it is a 3-fold NLP task [23]. The first subtask, called named entity recognition (NER), is to extract medical entities relevant to ADEs from text (such as medication, disorders, body parts, symptoms, etc); the second subtask, called relation extraction (RE), is to establish the relation between extracted entities (eg, a reason for prescribing a drug, causal relationship between an ADE and a drug, etc); and the last subtask (end-to-end integration) is to combine NER-RE models to decide whether an ADE is present in the given clinical text or not based on identified medical entities (NER model) and relations between them (RE model). NER and RE tasks can be implemented using two distinct approaches: (1) *c*lassical machine learning models (MLMs), such as support vector machines (SVMs), decision trees, conditional random fields (CRFs), and neural networks, among others; and (2) large language models (LLMs), such as Bidirectional Encoder Representations from Transformers (BERT; Google AI Language), Generative Pre-trained Transformer (GPT; OpenAI), RoBERTa (Meta AI), XLNet (Google Brain and Carnegie Mellon University), T5 (Google Research), among others.

The previous research results are difficult to compare because each study uses different entity, relation types, datasets, data sources (case reports, EHRs), and language (mainly in English) [15,24-29]. Nevertheless, using the MLM approach, researchers have achieved moderate accuracy in the NER task, with micro-average *F*_1_-scores reported at 0.80 [24], 0.82 [25], and 0.84 [15]. However, accuracy in the RE task remains low to moderate, with a macro-average *F*_1_-score of 0.28 [24] and a micro-average *F*_1_-score of 0.846 [25], due to the model’s limited contextual and semantic understanding of the text. The joined NER-RE tasks achieved a micro average *F*_1_-score of 0.869 [26]. Among those studies, only one study used clinical notes in the Swedish language [24], while all the others were in English. The LLMs achieved the highest accuracy using fine-tuned BERT on ADE detection in clinical notes in the English language (micro average *F*_1_-scores -0.93 for NER, 0.96 for RE task, and 0.895 for joined NER-RE [27] in relaxed match). The fine-tuned GPT 3.5 model achieved an *F*_1_-score of 0.70 (strict match) and 0.81 (relaxed match) [29] for the NER-RE (end-to-end) task in detecting ADEs in vaccine adverse event reports, and fine-tuned the Curie (GPT 3.5) model achieved 0.795 for NER and 0.74 for RE [28]. A recent study on classifying ADE reports in the Swedish language using GPT 3.5 achieved 0.71 *F*_1_-score [30]. Although the overall performance of the LLMs regarding NER and RE tasks is high, the micro-averaged *F*_1_-scores of each entity and relation type varied significantly. The lowest performance remains for detecting the ADE relations.

Our previous study on detecting ADEs in clinical notes using MLMs has achieved a micro average *F*_1_-score of 0.80 for NER and 0.28 for RE [24], showing a low performance in identifying relationships between medical entities. Unlike the MLM approach, using domain-specific variants of BERT allows the model to consider the full context of a word within a sentence, including distant dependencies and subtle linguistic nuances, which are crucial in accurately identifying and classifying entities in free-text medical records. In addition, BERT-based models benefit from pretrained embeddings that provide richer word representations in comparison with MLMs. Hence, even though transformer-based models like BERT are known to incorporate contextual information effectively, it remains critical to empirically evaluate their advantages on specific tasks such as ADE extraction from real-world EHR data. Such studies not only quantify performance improvements over traditional baselines but also identify persistent challenges, guiding further refinements in annotation schemes and model architectures.

Swedish Deidentified Clinical Bidirectional Encoder Representations from Transformers (SweDeClin-BERT) is a Swedish BERT model based on the KB-BERT model [31], which has been further pretrained on around 2 million deidentified and pseudonymized patient notes (17.9 GB) from over 500 clinical units at Karolinska University Hospital in Sweden [32]. This model showed good performance with 0.87 *F*_1_-score in the NER task, identifying entities belonging to sensitive information such as social security numbers, name, phone numbers, etc, for deidentifying clinical notes [33], *International Classification of Diseases, 10th Revision (ICD-10)* diagnosis coding with 0.94 *F*_1_-score [34], and classification triage of adverse drug reaction reports with 0.72 *F*_1_-score [35]. All these results show promising potential for enhancing RE performance for ADEs. This study aims to fine-tune the pretrained clinical language model, SweDeClin-BERT, for medical NER and RE tasks, and to implement an integrated NER-RE approach to more effectively identify ADEs in clinical notes from clinical units in Sweden. The performance of this approach will be compared to our previous machine learning method, which used conditional random fields (CRFs) and random forest (RF) [22].

## Methods

### Overview

Our study follows the JMIR study design category of “Machine learning predictive models in biomedical research”, and emphasizes a transparent, end-to-end approach—from data collection to model evaluation—aligning with best practices in reporting [36] to ensure clarity, reproducibility, and interpretation of model performance.

This study used transfer learning to approach 2 learning tasks, NER and RE, using the SweDeClin-BERT model. Transfer learning is the process of pretraining a model on a large-scale dataset and then reusing this model to improve performance on a related task [37]. This approach is particularly beneficial for our ADEs detection task, given the limited scale of annotated data and the complexity of free text, which poses significant challenges for machine comprehension. Encoder models like BERT are naturally suited to, and can be readily fine-tuned for, classification tasks, and SweDeClin-BERT has the advantage of having been adapted to the clinical domain. While it could be interesting to evaluate larger decoder models on these tasks, we consider this to be out of the scope of the current study. At present, there is no decoder mode that has been adapted to Swedish clinical text. Moreover, given the sensitive nature of the data, one would have to use an open-weight model that can be hosted in a secure on-premises environment with access to significant computational resources in order to allow for fine-tuning and inference. In this study, the performance of the fine-tuned SweDeClin-BERT models (NER and RE) is compared with our previous method as a baseline [24].

The baseline models are CRFs for the NER task and RF for the RE task used in our previous study [24] for identifying ADEs in clinical notes. The used CRF algorithm for the NER task was with a rich feature set, including token string, lemma, part-of-speech tags, capitalization, compound splitting, and terminology matching, along with distributional semantic features from various Distributional Semantic Models (DSMs) using different context window sizes. The used RF model for the RE task was with a hierarchical or cascading versus a flat classification approach. The method based on the hierarchical approach first classified the clinical notes into Containing a relation and No relation classes and then classified specific relation types. The feature set for the random forest classifier included one-hot encoded entity classes, entity unigrams and bigrams, the distance between entities, and context representation using multiple DSMs. Class imbalance was not addressed in the baseline approach. The issue of class imbalance, especially its effect on rare relation types such as ADE, ADE outcome, and ADE cause, is discussed in a previous study [24]. This limitation in the baseline design may affect the performance comparison and should be taken into account.

The following research question is addressed in this study: “How accurately can the clinical language model SweDeClinBERT detect ADEs in clinical notes compared to a traditional machine learning approach such as CRFs and RF?”

To answer this question, the pretrained SweDeClin-BERT model is fine-tuned for the NER and RE tasks, respectively, on the same dataset as the baseline model (395 annotated Swedish clinical notes called Stockholm EPR ADE Corpus), which was created in our previous study [24], and the results were compared to those of the baseline model. After the fine-tuning, 2 models (NER model and RE model) were stacked to obtain results for the integrated NER-RE task. In addition, the fine-tuned models (NER-RE pipeline) were evaluated on the note level with 2 classes: notes containing ADEs and notes without ADEs.

In order to compare the SweDeClin-BERT model performance with the baseline model, the dataset was divided once into training, validation, and test sets with splits of 70%, 10%, and 20%, respectively and used for all tasks: NER, RE, NER-RE, and ADE detection in clinical notes using NER-RE models. Grid search strategy [38] was used for hyperparameter tuning. Standard evaluation metrics such as Precision, Recall, *F*_1_-score, *F*_2_-score, and micro- and macro-averaged metrics were recorded for comparison with the baseline model.

Finally, we conducted a thorough model error analysis (for NER and RE models) by examining confusion matrices [39] and identifying terms most frequently misclassified. This analysis aims to guide future improvements in model training and input data preparation, helping to refine entity definitions and relationships, and ultimately enhance the model’s overall performance toward achieving the overarching goal. We carried out our experiments using Python 3, Jupyter Notebook, and spaCy library [40].

### Dataset

The training dataset Stockholm EPR ADE Corpus was obtained from the research infrastructure Health Bank [32] at Stockholm University. The clinical notes were originally sourced from Karolinska University Hospital, a large tertiary care facility in Sweden, and reflect documentation practices within the Stockholm regional health care system. The Stockholm EPR ADE Corpus (395 clinical notes, dated 2009‐2010) has previously been annotated by a physician for ADE-named entities (NEs) and the relations between them [24] and anonymized in this study, enabling us to evaluate the performance of the fine-tuned SweDeClin-BERT in identifying ADEs. A summary of the clinical notes used in the Stockholm EPR ADE Corpus is presented in Table 1. The dataset is heterogeneous and is not limited to a specific clinical domain. For more detailed information, please refer to our previous study [24].

**Table 1.** Description of the clinical notes used in this study.

Metric	Value
Total number of notes	395
Note categories	Physician notes from admission note, patient history, hypersensitivity and drug info, assessment, and discharge notes.
ICD-Codes	Notes with the following *International Classification of Diseases, 10th Revision *codes for ADE^a^: Z931, Y579, M819, M809, I952, T509, F050, T887, L271
Min character length	59
Max character length	4372
Mean character length	1014.78
Min words	7
Max words	645
Mean words	150.5
Mean number of sentences per note	14
Lexical diversity (avg. Type-token ratio [TTR])	0.70

a ADE: adverse drug event

The annotated NE categories include Drug, Disorder, Finding, Body Structure, and ADE Cue, where: Drug is a medication; Disorder is a not momentary disease or abnormal condition with underlying pathological processes; Finding is a symptom reported by the patient, observation by the physician, or a result from medical examinations, including a non-pathological finding with medical relevance; Body structure is an anatomical part of the body; ADE cue is an indicator of an ADE without specifying its form (eg, side effect, overmedicate, overdosage, medication reaction or respond, and hypersensitivity). This entity is used to indicate the presence of an ADE that is not explicitly described in clinical notes, where information about the drug or drugs associated with the ADE is missing.

Table 2 summarizes the number of instances (annotated entities) in each class within the Stockholm ADE Corpus. Notably, NEs can consist of multiple words, which complicates the NE task. For example, Findings and Disorders often consist of 2 or more words (called compound instances), making these classes harder to classify. ADE Cue, being a minority class with only 330 instances, presents particular challenges due to its limited representation. In addition, the average interannotator agreement (IAA) for the ADE Cue class is 0.53 (as reported in [24]), indicating significant difficulty in consistently classifying these entities.

**Table 2.** Overview of named entity classes and their instance counts before and after anonymization, including distribution in the test set. Percentages represent the proportion of each class relative to the total number of entities.

Entity class	Before Anonymization, n (%)	After Anonymization, n (%)	Number of instances in the test set
Finding	3184 (35)	3142 (35)	533 (3.2)
Drug^a^	2680 (29)	2613 (29)	493 (29.8)
Disorder^a^	1866 (20)	1848 (20)	367 (22.2)
Body structure	1132 (12)	1116 (12)	205 (12.4)
ADE cue	341 (3,7)	330 (4)	55 (3.3)
Total	9203 (100)	9049 (100)	1653 (100)

a There are some data discrepancies compared to previous study [24] due to data deidentification and data preprocessing.

According to the ADE definition by the World Health Organization (WHO), the clinical notes with complete or incomplete information about the associated drug or drugs indicate the presence of ADE [41]. Recognizing the cues of ADE with complete or incomplete information (missing information about the associated drug or drugs) is essential for comprehensive pharmacovigilance and patient safety. Therefore, the Stockholm ADE Corpus includes annotations for 4 types of relations covering cases with complete or incomplete information between the mentioned entity types as shown in Table 3: Indication, Adverse Drug Event (ADE), ADE Outcome, and ADE Cause. These relations are defined as follows:

- Indication: A relation from Finding or Disorder to Drug, indicating that the drug was prescribed as a result of the identified Finding or Disorder.
- ADE: A relation from Drug to Finding or Disorder, specifying that a Finding or Disorder developed after taking the medication.
- ADE Outcome: A relation from ADE Cue to Finding or Disorder, highlighting the occurrence of the ADE without specifying its underlying cause.
- ADE Cause: A relation from Drug to ADE Cue, identifying that the drug is responsible for an ADE, without detailing the nature of the ADE.
- No Relation: is introduced after anonymization to represent unrelated entity pairs

**Table 3.** Overview of relation classes and their instance counts before and after anonymization, including distribution in the test set.

Relation class	Before anonymization, n (%)	After anonymization, n (%)	Number of instances in the test set, n (%)
Indication	1392 (53)	1203 (4)	220 (3.7)
Adverse Drug Event	855 (37)	810 (3)	156 (2.7)
ADE Outcome	144 (5)	139 (0.5)	23 (0.4)
ADE Cause	228 (9)	221 (0.7)	38 (0.6)
No relation	—^a^	28,412 (92)	5391 (92.5)
Total	2619 (100)	307,85 (100)	5828 (100)

a —: not applicable.

In essence, ADE Outcome and ADE Cause provide incomplete information about adverse drug reactions. These 2 classes are the least common relations in the corpus, with only 139 and 221 instances, respectively.

The dataset contains clinical notes as text files (in .txt format), anonymized annotated notes with BIO Schema tags [42] files (in .CoNLL format), and relation annotations files in Brat [40] format (.ann files). In BIO Schema, B indicates the beginning of an entity, I denotes tokens that are part of the same entity, and O marks tokens that do not belong to any entity (category Other). Specifically, each token is tagged with one of the following 11 labels: O, B-Finding, I-Finding, B-Drug, I-Drug, B-Disorder, I-Disorder, B-Body Structure, I-Body Structure, B-ADE Cue, and I-ADE Cue. Brat is an open-source web-based tool for text annotation [43] and was used by annotators in our previous study.

### Missing Data and Data Cleaning

There were 5 empty notes out of 400; thus, the final number of notes is 395. Data cleaning involved filtering out notes without entities; however, since all notes contained entities, the dataset remained at 395 notes.

### Outcome and Variables

The outcome of the NER task is to detect the ADE-related entities, including Disorder, Finding, Drug, Body structure, and ADE cause, and the outcome of the RE task is to correctly classify the ADE relation between the identified entities. For the integrated NER-RE task, the outcome is to determine whether a clinical note contains a description of an ADE or not (yes or no).

For both the NER and RE tasks, the input to the SweDeClinBERT models consists of tokenized clinical text segments. The text is preprocessed and encoded using SweDeClinBERT’s tokenizer, which splits the input into subword tokens and converts them into numerical embeddings that capture contextual meaning. This embedding is a numerical vector of variables, where each dimension captures distributed and context-dependent information, rather than corresponding to individual words, reflecting the semantic structure of the entire input.

### Sample Size

The sample size in this study is fixed, consisting of 395 anonymized clinical notes, and is not intended to be representative of the broader patient population. Rather than supporting statistical generalization, this study is exploratory in nature. Its primary aim is to evaluate and compare the performance of a domain-specific NLP model (SweDeClinBERT) against a traditional MLM (conditional random fields) in the task of ADE detection.

### Named-Entity Recognition Task

**Figure 1.**
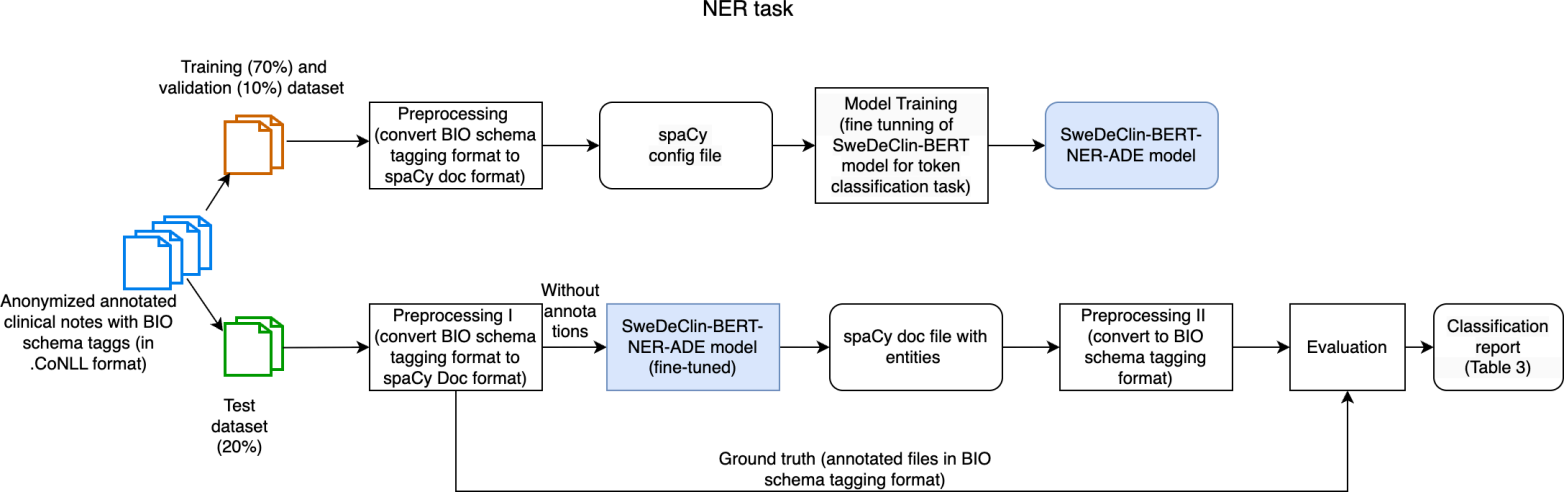
Workflow of the named entity recognition task for extracting adverse drug event-related entities from clinical notes. NER: named entity recognition; NER-ADE: named entity recognition-adverse drug event; SweDeClin-BERT-RE-ADE: Swedish Deidentified Clinical Bidirectional Encoder Representations from Transformers-named-entity recognition of adverse drug event;

The NER task pipeline consists of training (fine-tuning as a token classification task) and evaluation phases, as shown in Figure 1.

In the training phase, the texts were preprocessed by converting BIO Schema tagging to spaCy Doc type, which is suitable for model training. In addition, texts were segmented into smaller, overlapping chunks to accommodate the processing of longer texts that exceed the model’s maximum input length. Each chunk was limited to 128 tokens in length, with a stride of 96 tokens. The spaCy config file is a file containing the configuration needed for fine-tuning the SweDeClin-BERT model. The SweDeClin-BERT model was fine-tuned for the NER task as a token classification task. During fine-tuning, BERT’s output layer is modified to predict the appropriate tag for each token based on the contextual embeddings produced by the model. The different hyperparameters that were tuned are shown in Table 4.

**Table 4.** Hyperparameters used for fine-tuning the named entity recognition model.

Hyperparameter	Value
Batch size	128
Dropout rate	0.1
Optimizer	Adam
Learning rate	0.00005
Gradient accumulation	3
Gradient clipping threshold	1.0
Early stopping	With a patience value of 1600 steps
Weight decay	With L2=0.01

In the evaluation phase, the fine-tuned SweDeClin-BERT-NER-ADE model was used in prediction mode (input without annotations) to predict the entities. Afterward, the output of the SweDeClin-BERT-NER-ADE model (predicted entities) was converted back to BIO Schema tags format for measuring model performance with the ground truth (see classification report in Table 3).

### Relation Extraction Task

**Figure 2.**
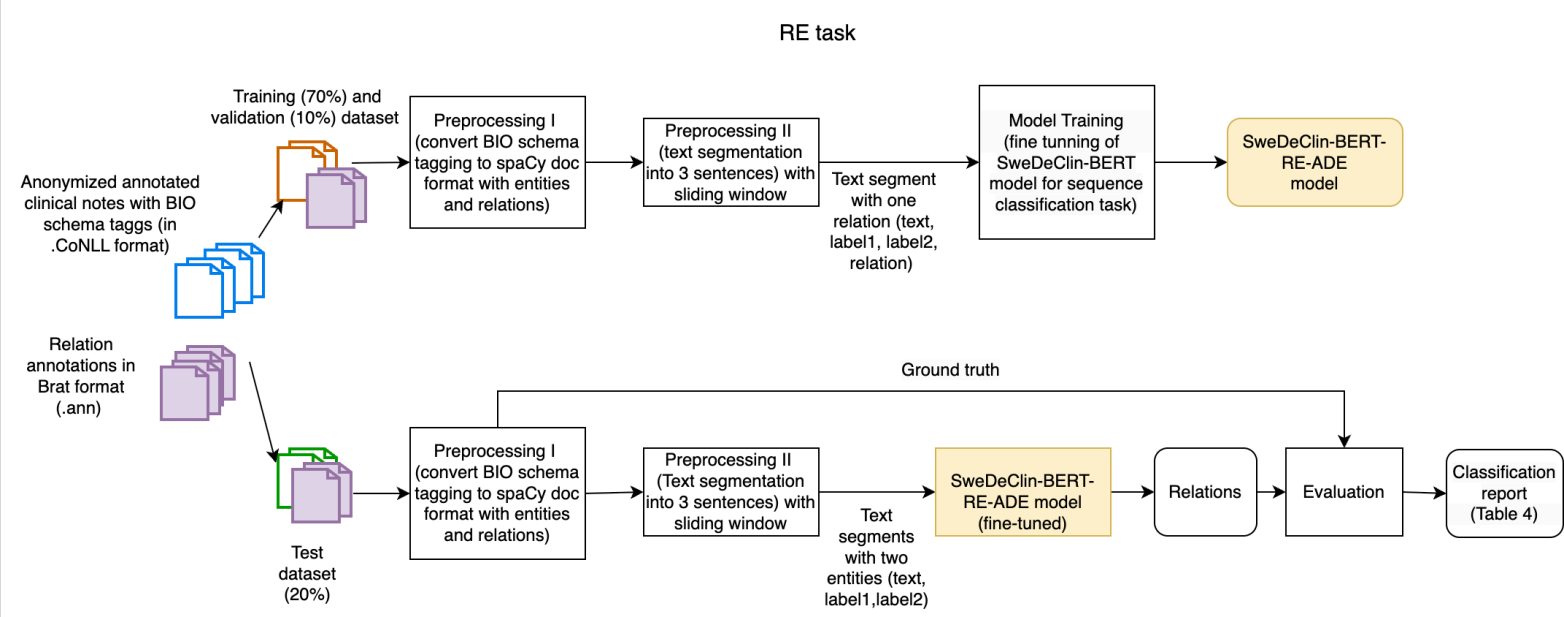
Workflow of the relation extraction task for identifying relationships between adverse drug event-related entities. RE: relation extraction; SweDeClin-BERT-RE-ADE: Swedish Deidentified Clinical Bidirectional Encoder Representations from Transformers-relation extraction-adverse drug event;

Similarly to NER, the RE pipeline also consists of training (fine-tuning a sequence classification task) and an evaluation phase as shown in Figure 2.

Given that the performance of the RE model is even more dependent on the context, preprocessing the texts for this task was crucial in the training phase. Since the maximum length processed by SweDeClin-BERT is 512 tokens, and relations between entities in the annotated texts often span no more than three sentences, the input texts were segmented into shorter passages of three sentences each, with a stride of one sentence. This segmentation preserved the contextual information necessary for accurate relation extraction while managing computational complexity effectively. The input to the model is a text segment containing only two entities and the relation between them. In addition to the primary relation classes of interest (Indication, ADE, ADE Cause, and ADE Outcome), a No relation class was added. This class was assigned to specific entity pairs where these primary relations could potentially exist but were not annotated. As shown in Table 3, the No relation class constitutes the majority class. The different hyperparameters that were tuned are shown in Table 5. After fine-tuning the SweDeClin-BERT model for the RE task, the best model was saved as the SweDeClin-BERT-RE-ADE model.

**Table 5.** Hyperparameters used for fine-tuning the extraction model.

Hyperparameter	Value
Batch size	32
Epochs	50
Patience for early stopping	3
Optimizer	Adam
Loss	Cross-entropy
Learning rate	2.575e-05
Weight decay	1e-4

In the evaluation phase, the fine-tuned SweDeClin-BERT-RE-ADE model was used in prediction mode (with annotated entities but without annotated relations) to predict the relations between 2 entities. Afterward, the SweDeClin-BERT-RE-ADE model performance was evaluated using the ground truth (see classification report in Table S1 in Multimedia Appendix 1) [24].

### Integrated NER-RE Task

**Figure 3.**
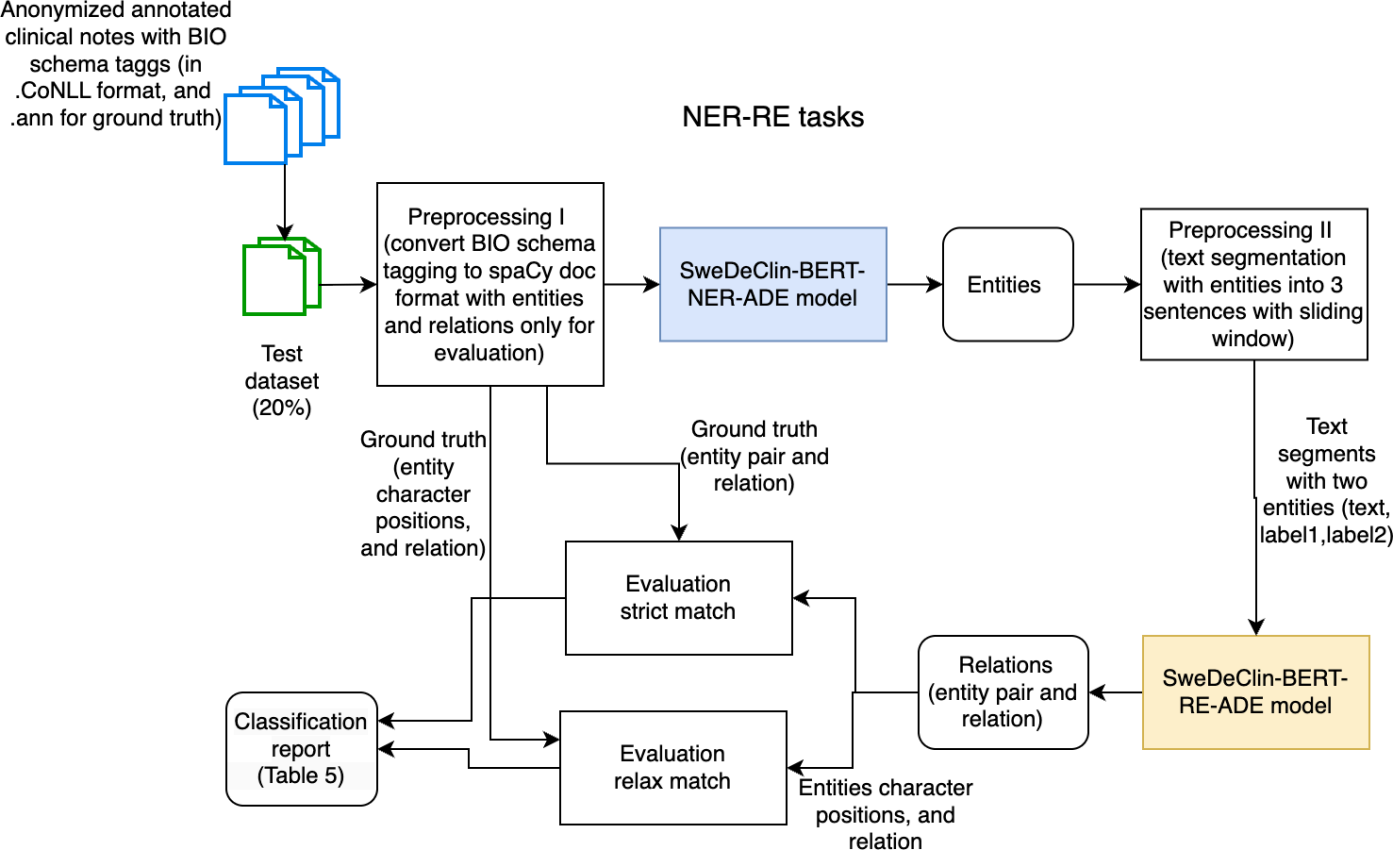
Integrated workflow combining entities and relation extraction tasks for adverse drug event detection on entity pairs. NER-RE: named entity recognition-relation extraction; SweDeClin-BERT-RE-ADE: Swedish De-identified Clinical Bidirectional Encoder Representations from Transformers-relation extraction-adverse drug event

Two fine-tuned models (NER-model and RE-model) were stacked to obtain results for the integrated NER-RE task, as shown in Figure 3.

The SweDeClin-BERT-NER-ADE model first classified entities within the text, and these entities were then preprocessed to be used by the SweDeClin-BERT-RE-ADE model to establish relationships. SweDeClin-BERT-RE-ADE model accepts a text segment with only 2 entities. This sequential stacking of models introduces the challenge of error propagation. Specifically, any entity missed by the SweDeClin-BERT-NER-ADE model would not be considered for relation classification by the SweDeClin-BERT-RE-ADE model, leading to unidentified relations. In addition, misclassifications at the NER stage could result in the incorrect exclusion of potential relations due to predefined rules. For example, if an entity labeled as a Finding was misclassified as a Body Part, the system would ignore potential relationships involving this misclassified entity, as certain relations (such as between a Drug and a Body Part) were deemed invalid.

The proposed integrated NER-RE task was evaluated using 2 modes: strict and flexible matching. The strict match required an exact match for all entities, including multiword entities, while the flexible match allowed for partial matches, recognizing cases where at least one word from a multiword entity was correctly identified.

### ADEs Detection in Clinical Notes Using Integrated NER-RE Pipeline

**Figure 4.**
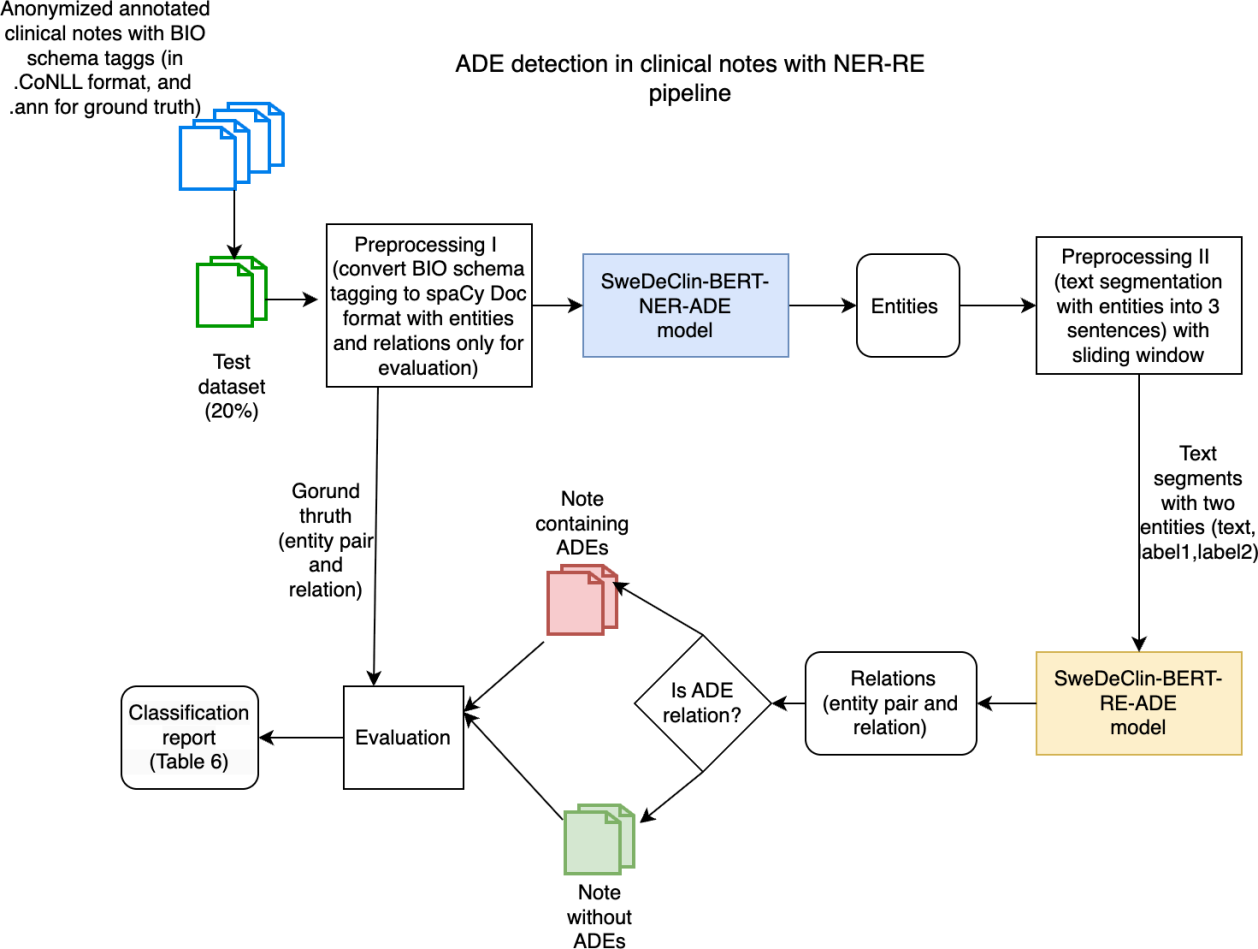
Adverse drug event detection in clinical notes using the integrated pipeline of named entity recognition and relation extraction models. ADE: adverse drug event; NER-RE: named entity recognition-relation extraction; SweDeClin-BERT-RE-ADE: Swedish Deidentified Clinical Bidirectional Encoder Representations from Transformers-relation extraction-adverse drug event

Given the overarching goal of identifying whether a text contained information about an ADE, we also evaluated the system’s ability to classify texts as either containing ADE information or not (Figure 4).

This binary classification was implemented as a rule-based decision based on the predictions from the integrated NER-RE pipeline. The rule-based decisions were made whether at least one ADE relation was present in a clinical note and then labeled as a note containing ADE. This evaluation metric helped in understanding the practical applicability of the model in finding clinical notes with potential ADEs.

### Ethical Considerations

This research has been approved by the Regional Ethical Review Board (Etikprövningsnämnden), permission number 2012/834-31/5 and the Swedish Ethical Review Authority (Etikprövningsmyndigheten), permission number 2023-06920-01. The original consent approval covers secondary analysis without additional consent. All data used in this study are anonymized (do not contain any private information, like names, phone numbers, address, etc).

## Results

### NER Task Results

In evaluating the NER task on the holdout data (test dataset), our approach using SweDeClin-BERT demonstrated superior performance across most entity categories (see Table S2 in Multimedia Appendix 1) compared with the baseline model [24].

For the key entities related to ADE, including Finding, Disorder, and Drug, our approach outperformed the baseline. Specifically, for the entity category Finding, our model achieved an *F*_1_-score of 0.802, compared to the baseline’s 0.71. This improvement is due to balanced enhancements in both precision (0.798 vs 0.756) and recall (0.807 vs 0.67). In the Disorder category, our model recorded an *F*_1_-score of 0.859, surpassing the baseline’s 0.811, with superior precision (0.865 vs 0.83) and recall (0.853 vs 0.793). The Drug entity category also showed gains, with our model reaching an *F*_1_-score of 0.946, compared to the baseline’s 0.886.

### RE Task Results

Evaluation results using fine-tuned SweDeClin-BERT for the RE task (SweDeClin- BERT-RE-ADE) on the holdout data revealed improvements across all relation categories (see ) over the baseline results. Specifically, for the Adverse Drug Event category, our model attains an *F*_1_-score of 0.704, compared to the baseline’s 0.146.

Overall, the macro-average scores reflect improvements, with our model achieving an *F*_1_-score of 0.813, in sharp contrast to the baseline’s 0.281 *F*_1_-score. These results strongly suggest that transformer-based architectures outperform traditional machine learning approaches in relation extraction, likely due to their superior ability to capture contextual information.

### NER-RE Integrated Task Results

The evaluation results for the integrated task (NER-RE) for Strict and Relaxed matches are presented in Table S3 in Multimedia Appendix 1. In this table, the N column represents the total number of holdout instances in each category in the test dataset, and the Classified column refers to the number of instances for which the model predicted a relation.

Overall, the NER-RE pipeline performance slightly decreased, as expected, because of error propagation from the NER model. However, looking at the macro average scores in Table S3 in Multimedia Appendix 1, the NER-RE pipeline performed well in both strict and relaxed match modes (0.815 and 0.812 *F*_1_-score, respectively). The obtained results show that all relation categories obtained slightly higher performance in relaxed match mode, although the differences between the 2 modes in performance are next to negligible.

### ADE Detection in Clinical Notes

The evaluation results of ADE detection in clinical notes are shown in Table S4 Multimedia Appendix 1. Specifically, in the relaxed matching mode, the model achieved an *F*_1_-score of 0.828 for Containing ADE class, and 0.789 for the No ADE class.

Overall, the macro-average scores across both categories are moderate (0.771 in strict and 0.808 in relax matching). These results highlight the model’s balanced performance in ADE detection, effectively identifying most ADE cases while maintaining moderate accuracy for non-ADE instances (*F*_1_-score with 0.789).

Overall, only 62% (246 clinic notes) contain explicit description of the ADEs.

### Error Analysis

#### NER Task Errors

The error analysis for NER model reveals several key insights into misclassification patterns. Specifically, the ADE Cue class emerges as the most frequently misclassified category (see Table S5 inMultimedia Appendix 1), with numerous instances being erroneously labeled as Other or Disorder. Notably, 13 (in comparison to 37 true positives) instances were classified as Other, indicating a lack of sufficient examples to accurately identify entities of this class in the corpus. In addition, 7 instances were misclassified as Disorder, with token examples such as “excess substitution,” “allergy,” “reaction,” “toxic,” “skin side effects,” and “exanthem.”

It is important to note that the terms “allergy,” “reaction,” and “exanthem” frequently appear within compound entities (consisting of 2 or more words) that are annotated under three different categories: ADE Cue, Disorder, and Finding. This observation suggests that the gold standard annotations for these classes may need refinement to improve entity recognition performance.

The second most misclassified category is Finding, with 132 instances (in comparison to 581 true positives) incorrectly labeled as Other. It’s worth noting that Finding has the highest number of compound instances, accounting for 23% (718 out of 3142) of all Finding instances in the corpus. Among the instances misclassified as Other, we observed terms such as “weak,” “general,” “new symptoms,” “problem,” “histamine release,” “healthy,” “improvement,” “positive,” “worsened,” “progressed,” “prothrombin time,” “good condition,” “borrelia,” and “skin flora.” In this list, we see examples of findings without specificity, such as problem and new symptoms, which might appear in contexts other than medical findings. This could be attributed to the pretraining of SweDeClin-BERT on the general-purpose KB-BERT model. On the other hand, the list also includes instances that were simply missed by the model, such as critical findings like histamine release, borrelia, and prothrombin time.

For compound instances where only one part of the instance was tagged as Finding, we can mention low-grade nausea, with only nausea (illamående) classified as Finding*;* general malaise, with only malaise (illabefinnande) classified as Finding; and increased infection susceptibility, with only infection susceptibility (infektionsbenägenhet) labeled. This suggests that our model struggles with compound terms, particularly those with detailed descriptions. It often recognizes only the core component (the primary finding) of the entity while missing the additional descriptive elements that provide context. This limitation underscores the need for further refinement of the annotation guidelines or additional training data.

In the Disorder category, the majority of misclassifications occur as Finding (33 instances) and Other (18 instances). Misclassifications of Disorder entities as Other include examples like fungus (svamp) and malignancy (malignitet). In addition, there are compound cases where only one part of the entity is correctly labeled. For example, in partial cortisol deficiency (partiell kortisolbrist), the term cortisol deficiency is labeled as Disorder; in acute hives (akut urtikaria), hives are labeled as Disorder; and in secondary infection (sekundär infektion), infection is labeled as Disorder. There are also instances where Disorder entities are misclassified as ADE Cue, such as drug-induced rash (läkemedelsexanthem, läkemedelsutslag), a term that appears in both the Disorder and ADE Cue categories in the gold standard.

In the Drug category, some instances were misclassified as Findings or ADE Cues. For example, requiring oxygen (syrgaskrävande) was misclassified as a Finding, and Claforan-induced (Claforanorsakat) was misclassified as an ADE Cue. In these cases, the issue seems to be related to inconsistencies in the gold standard rather than the model’s performance. In addition, some instances were misclassified as Other, such as medication (läkemedel, medicin), where this term appeared multiple times in the text, with the model only labeling one instance correctly.

Body Structure is the least misclassified category, with only 2 terms in compound instances misclassified as Findings and 6 instances misclassified as Other.

#### RE Task Errors

Examining the errors in the RE step, as detailed in Table S6 in Multimedia Appendix 1, we observe that the RE model primarily misclassifies relation class instances as No relation. Specifically, out of 199 classified Indication instances, 36 were misclassified as No relation. Similarly, out of 136 ADE instances, 46 were misclassified as No relation. In the ADE Outcome category, 3 out of 15 instances were misclassified as No relation, and out of 30 ADE Cause instances, 5 were misclassified as No relation.

While these errors are notable, they do not provide new insights due to the predefined rules governing permissible relations. These rules limit the possibility of misclassifying entities across the 4 primary relations of interest (Indication, ADE, ADE Outcome, and ADE Cause). However, there are other types of errors that are worth mentioning. For example, 2 out of 30 ADE Cause instances were misclassified as ADE.

This error appears to stem from mistakes at the NER stage, particularly in labeling ADE Cues as Finding or Disorder.

Looking into the specifics of the misclassifications, particularly for the ADE class, we find that very general reactions are often misclassified as ADEs, such as itching (klåda), swelling (svullnadskänsla), nausea (illamående), allergic reaction (allergisk reaktion), rash (utslag), anxiety (oro), and pain (värk). Some of these terms appear in the list of relations misclassified as Indication, such as pain (värk), swelling (svullnadskänsla), and allergic reaction (allergisk reaktion).

Among the specific medications in ADE relations misclassified as No relation instances, we found Digitalis, Enalapril, Seretide, Symbicort, antihistamine, asthma medication, botox, docetaxel, and epirubicin.

Overall, these errors suggest that important ADEs are being missed by the model, and increasing the training data could potentially improve classification.

## Discussion

### Principal Findings

This study investigated whether the LLMs approach, like the SweDeClin-BERT model, allows better detection of ADEs in clinical notes in comparison to the MLMs approach, like conditional random fields and random forest. We successfully fine-tuned the SweDeClin-BERT model for medical NER and RE tasks to identify ADEs in Swedish clinical notes. The integrated NER-RE pipeline achieved an overall *F*_1_-score of 0.81, with the NER task reaching 0.845 and the RE task 0.813. These results significantly outperform our previous machine learning approach using CRFs and random forests [24], with a 5% improvement in NER and a 53% gain in RE macro-average *F*_1_-score. These findings confirm that a transformer-based model provides a more effective approach for ADE identification in clinical text.

Only 62% of notes contained an explicit description of ADE, which indicates that the presence of an ADE-related ICD code does not guarantee that the clinical note contains detailed information about the event.

Despite the moderate performance (0.815 *F*_1_-score) of our integrated NER-RE pipeline, the *F*_1_-scores for individual entity classes in the NER task vary. For instance, the ADE Cue entity class has the lowest performance, with an *F*_1_-score of 0.680. This is due to unbalanced entities in the NER task in the dataset. The unbalanced issue can be addressed by increasing the dataset with more ADE notes. Another possible explanation for the low accuracy in the ADE Cue entity in the NER task is that the annotators’ agreement is low (0.53), indicating that there are some refinements in the data annotations that should be applied (better consistency).

Summarizing the results of the error analysis for the NER task, we identify several key issues: lack of sufficient examples to accurately identify entities of the ADE Cue class in the corpus; overlap of words between the three categories: ADE Cue, Disorder, and Finding; multistem words with stems belonging to different entities but tagged as one entity; general terms that sometimes belong to entities (eg, besvär, problem) and other times do not; inconsistent tagging of repeated mentions of the same entity (eg, läkemedel mentioned several times). Most of these issues are related to the issue with compound and multistem words, which are very common words in clinical text in the Swedish language. A possible suggestion for dealing with compound and multi-stream words could be to address this during the annotation process (annotate the subword of a word separately). This will allow the SweDeClin-BERT model to handle unseen or rare compound words by breaking them into known subwords, which helps in maintaining the semantic meaning.

We also observe differences in the performance of the RE model across various class categories. Despite having more examples for the classes of primary interest, Indication and ADE, compared to ADE Outcome and ADE Cause, the RE model performs better in classifying the latter, with *F*_1_-scores of 0.810 and 0.816 for ADE Outcome and ADE Cause, compared to 0.757 and 0.704 for Indication and ADE. This higher performance can be attributed to the presence of ADE Cue words in relations ADE Outcome and ADE Cause. As reported in [24], ADE Cues consist of a relatively short set of words (130 terms) compared to the more varied Disorders and Findings, which have 976 and 1533 unique words, respectively. This suggests that there may still be insufficient training data to cover all the diverse contexts of the ADE relation.

### Comparison With Previous Work

In comparison, previous studies on ADE detection in clinical notes using LLMs, although based on clinical texts in English, achieved higher accuracy for the NER and RE tasks (with the microaverage *F*_1_-scores at 0.93 and 0.96, respectively [27]). However, the performance of the integrated NER-RE pipeline reported in previous studies was comparable to that of our study. For instance, the previous NER-RE pipeline achieved a micro average *F*_1_-score of 0.895 in [27], and micro *F*_1_-scores of 0.70 for strict matching and 0.81 for relaxed matching in [29]. In comparison, our study reports macro average *F*_1_-scores of 0.815 for strict matching and 0.812 for relaxed matching. We use a macro average statistic to account for the presence of the dominant No relation class in our data.

### Limitations

This study has several limitations that impact the generalizability of the results. First, the dataset was annotated by a single physician and 2 nonclinicians, with low interannotator agreement observed across key entity types. This raises concerns about the consistency and reliability of the gold standard annotations, which in turn may have limited the achievable model performance. Disagreements among annotators, particularly between clinical and nonclinical raters, suggest that the annotation guidelines may not have been sufficiently robust for nonexpert interpretation.

Second, the dataset’s relatively small size (395 clinical notes) and the unbalanced distribution of ADE classes constrain the model’s ability to generalize across broader clinical settings. It may introduce selection bias, as the dataset overrepresents ADEs compared to their real-world prevalence. Annotation bias may arise from subjective interpretation of guidelines, and temporal bias may limit the generalizability of the model to other time periods. Although this dataset was created over a decade ago with some known limitations in annotation quality, it provides a valuable opportunity for longitudinal comparison. Previous work relied on traditional machine learning methods such as CRF, which predated the development of transformer-based models like BERT. Reapplying modern deep learning techniques to the same clinical text enables us to assess how methodological advances can improve ADE extraction, even when working with imperfect data.

Third, the rule-based approach used in our current study is limited in its ability to capture deeper contextual nuances such as temporality, negation, speculation as in our previous study [24], and attribution. These factors are crucial for accurately detecting ADEs in clinical notes, as ADE mentions may refer to past or future events, be negated or speculative, or refer to individuals other than the patient (eg, a family member mentioned in the patient’s medical history). Furthermore, this study did not investigate variations in language use, documentation styles across different clinics, or shifts in ADE prevalence. Our current approach does not capture this level of contextual information and leaves it as an important direction for future research.

### Summary

To summarize, the results demonstrate that fine-tuned SweDeClin-BERT models for NER and RE are effective for detecting ADEs in Swedish clinical notes when used in an integrated pipeline. These findings suggest that transformer-based models can serve as valuable tools for augmenting pharmacovigilance efforts by uncovering ADEs not captured by structured EHR data alone. However, improved handling of compound words and refinements to entity annotations remain important to increase accuracy and reduce ambiguity. In particular, merging “Disorder” and “Finding” into a single “Finding” class and excluding the “BodyPart” class are expected to simplify the schema and enhance annotation consistency, as already evidenced in our ongoing work.

### Conclusions

Using a domain-specific language model like SweDeClin-BERT for detecting ADEs in Swedish clinical notes enables moderately accurate identification, achieving an *F*_1_-score of 81%. This approach also improves relation extraction accuracy by 53% compared to our previous random forest method. Key challenges in classifying ADE Cue entities have been identified and will be addressed in our upcoming study. Detecting ADEs in clinical notes is indeed a challenging task, and more research is needed to be able to use the valuable information documented in free text instead of being documented in a structured manner. The integrated NER-RE pipeline, powered by fine-tuned SweDeClin-BERT models, offers the potential to identify suspected ADEs in clinical notes that are not explicitly reported in EHRs.

## Supplementary material

10.2196/71949 Multimedia Appendix 1 Performance metrics (precision, recall, and F1) for SweDeClin-BERT-ADE compared with baseline CRF

## Abbreviations

ADE: adverse drug event
AI: artificial intelligence
BERT: Bidirectional Encoder Representations from Transformers
BIO: Begin Inside Outside
CRF: Conditional Random Fields
DSM: Distributional Semantic Models
EHR: electronic health record
GPT: Generative Pre-trained Transformer
ICD-10: International Classification of Diseases
LLM: large language models
NER: named-entity recognition
NLP: natural language processing
RE: relation extraction
RF: random forest
SweDeClin-BERT: Swedish Deidentified Clinical Bidirectional Encoder Representations from Transformers
WHO: World Health Organization
